# An Interesting Case of Peripartum Cardiomyopathy With Biventricular Thrombi

**DOI:** 10.7759/cureus.38748

**Published:** 2023-05-09

**Authors:** Joseph Abi Jaoude, Alyssa Golden-Hart, Greg Stanger, Mariam Hashmi, Kipson Charles, Liang Sun, Matthew Calestino

**Affiliations:** 1 Internal Medicine, University of Central Florida/HCA Florida Healthcare GME Consortium, Gainesville, USA

**Keywords:** congestive hepatopathy, ventricular thrombosis, peripartum cardiomyopathy, cardiomyopathy, acute heart failure

## Abstract

Peripartum cardiomyopathy (PPCM) is a cause of heart failure that develops within five months postpartum. Biventricular thrombosis is a rare complication of PPCM with only a few cases reported in the literature. Here, we report a case of PPCM with biventricular thrombosis that was successfully treated with medical management.

## Introduction

Peripartum cardiomyopathy (PPCM) is a rare cause of heart failure that develops in the last month of pregnancy or within five months postpartum [1,2]. Although limited data on PPCM rates exist, the incidence of PPCM seems to range between one in 4,000 and one in 300 [3,4]. Many contributory factors including autoimmune and viral are associated with the development of PPCM; however, no definite etiology for PPCM has been identified [5]. The prognosis of PPCM is overall favorable, with around 70% of patients recovering cardiac function by six months [6]. Ventricular thrombosis, and particularly biventricular thrombosis, is a rare complication of PPCM, with only a few cases reported in the literature [7]. Here, we report a case of PPCM with biventricular thrombosis.

## Case presentation

A 32-year-old female, G7P4A3, with a past medical history of asthma, hypertension, and class II obesity presented with worsening dyspnea over the last three to four months. The patient was five months postpartum from induction of labor with normal spontaneous vaginal delivery due to chronic hypertension with superimposed pre-eclampsia with severe features. Around one to two months after delivery, the patient started developing progressive dyspnea that worsened on exertion and lying flat. The patient also noted having new-onset bilateral lower extremity edema, 5 kg weight gain, and new-onset epigastric and suprapubic pain. The patient was referred to the Emergency Department (ED) after these symptoms were noted during a visit to her primary care physician.

**Figure 1:**
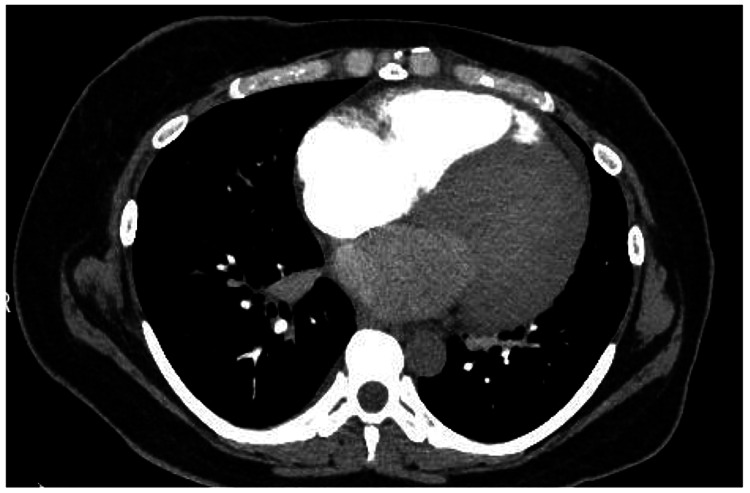
CT of the chest with intravenous contrast showing cardiomegaly.

In the ED, the patient seemed to be in respiratory distress with mild tachypnea. Electrocardiography (EKG) showed sinus tachycardia and arterial blood gas analysis showed respiratory alkalosis (pH = 7.48, pCO_2_ = 31, pHCO_3_ = 23.5) on a nasal cannula (FiO_2_ = 28%). On labs, the patient was noted to have elevated pro-brain natriuretic peptide (3,975 pg/mL), aspartate aminotransferase (321 U/L), and alanine transaminase (545 U/L) (Table 1). A CT angiography of the chest was negative for pulmonary embolism but showed mild cardiomegaly with small-to-moderate pericardial effusion (Figure 1). A CT of the abdomen and pelvis showed mild-to-moderate diffuse ascites of unclear etiology, with heterogeneous enhancement of the liver. The patient was admitted to the Cardiovascular Intensive Care Unit for PPCM with congestive hepatopathy, and the cardiology, OB-GYN, gastroenterology, and palliative teams were consulted.

**Table 1:** Lab values at admission and discharge. ALP: alkaline phosphatase; ALT: alanine transaminase; AST: aspartate transaminase; BUN: blood urea nitrogen; Hb: hemoglobin; Hct: hematocrit; Pro-BNP: brain natriuretic peptide; WBC: white blood cells

Lab value	Admission	Discharge
Sodium	138	139
Potassium	4.2	3.7
Chloride	109	103
Bicarbonate	20	25
BUN	33	29
Creatinine	1.09	1.21
Calcium	8.6	8.6
Magnesium	2.5	2.7
Troponin	17	-
Pro-BNP	3,975	-
AST	321	49
ALT	545	146
ALP	119	114
Total bilirubin	1.2	0.6
Total protein	6.9	7.2
Albumin	2.9	3
Lipase	115	-
WBC	12.5	12.1
Hb	10.6	13.1
Hct	34.8	42.9
Platelets	566,000	511,000

**Figure 2:**
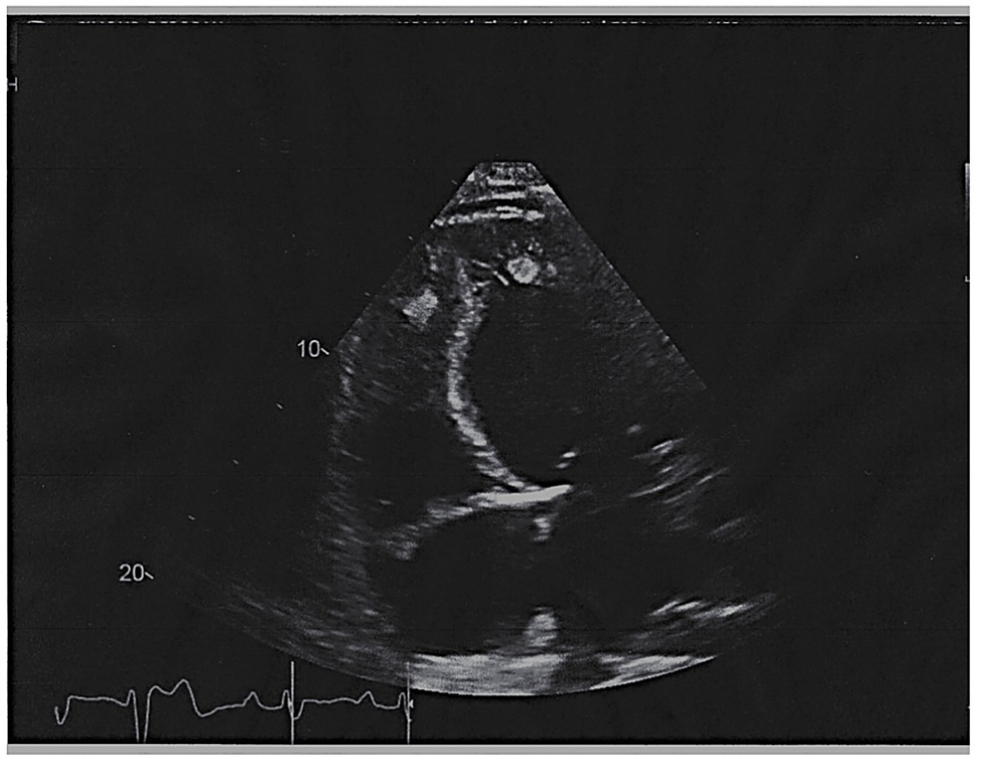
Echocardiogram showing cardiomyopathy with biventricular thrombi.

The patient was started on intravenous (IV) furosemide and milrinone, and a Foley catheter was inserted for accurate in/out measurements. During her stay, the patient developed non-sustained ventricular tachycardia, for which she was started on amiodarone. A transthoracic echocardiography (TTE) was ordered, and the patient was scheduled for cardiac catheterization. TTE was performed and showed severe biventricular systolic dysfunction with a severely dilated left ventricle (ejection fraction of 15-20%) and biventricular hyperechoic fixed thrombi (Figure 2). The patient was then started on an IV heparin drip for her thrombi. Three days later, milrinone was discontinued, and the patient was started on carvedilol and sacubitril/valsartan. On day four, the patient underwent cardiac catheterization that showed non-ischemic cardiomyopathy with widely patent coronaries and no evidence of obstructive coronary artery disease. A left ventriculogram was not performed due to a left ventricle thrombus.

After cardiac catheterization, the patient was started on metoprolol 12.5 mg twice daily, dapagliflozin 10 mg daily, and heparin was transitioned to apixaban. The hematology team was consulted and recommended continuing anticoagulation for at least six months, with follow-up as an outpatient. The patient was hemodynamically stable and her condition and labs improved (Table 1). She was discharged in stable condition on sacubitril/valsartan, furosemide, metoprolol, apixaban, dapagliflozin, and amiodarone, and was scheduled for cardiac MRI and follow-up with cardiology and hematology. The patient was advised that breastfeeding would be contraindicated and that she should strongly consider contraception owing to the risks associated with her medical condition if a future pregnancy is to happen.

## Discussion

PPCM is a rare disease that occurs in the last month of pregnancy or within five months postpartum and presents with clinical manifestations of heart failure [8]. Ventricular thrombosis is a rare complication of PPCM, with only a few cases reported in the literature, and even fewer cases of biventricular thrombosis reported in the literature [9,10]. We report a case of PPCM that developed approximately one to two months postpartum. After examination with echocardiography, the patient was found to have a severe decrease in ejection fraction and biventricular thrombi.

While many theories exist, the exact etiology of PPCM remains unknown. Myocarditis has often been suggested as a cause for PPCM, mainly because of the common presence of inflammatory infiltrates evident on endomyocardial biopsies or cardiac MRI [11]. Another possible reason for the development of PPCM is the triggering of autoimmunity in the mother. This occurs through chimerism, where fetal-derived cells cross into the maternal circulation and eventually settle in the mother’s heart [12]. This theory is further supported by the evidence that PPCM is associated with high titers of autoantibodies against multiple cardiac tissue proteins [12]. Other potential causes for PPCM that have been proposed include iron deficiency, selenium deficiency, prolonged tocolysis, stress-activated inflammatory cytokine release, and abnormalities in the ovarian hormone relaxin [12,13]. Interesting to note is that despite the many hemodynamic changes that occur during pregnancy, those do not seem to be related to PPCM [13]. This is evident owing to the timing of PPCM, where most hemodynamic changes occur during the first and second trimesters, while PPCM is vastly a disease of the third trimester or postpartum period [13].

Ventricular thrombosis is a rare complication of PPCM. Nevertheless, owing to the hypercoagulable state that occurs during pregnancy, PPCM is associated with higher rates of thromboembolism compared to other forms of cardiomyopathy [14]. Multiple factors contribute to the hypercoagulable state in pregnancy, including cardiac dilation, endothelial injury, increased clotting factor production, and immobility [13]. As such, anticoagulation can be considered in patients with PPCM during pregnancy and up to two months postpartum. While some form of thromboembolism can be expected with PPCM, the development of biventricular thrombi is very rarely reported in the literature. Similar to our patient, most cases of biventricular thrombosis reported in the literature were treated with anticoagulation therapy, as surgical resection may exacerbate cardiac function [7]. It is important to note that thromboembolism in PPCM is not always limited to the cardiac ventricles, but has also been reported in the cerebral vasculature, coronary arteries, and splenic arteries [15-17].

Very limited data exist on the treatment of patients with PPCM, and as such, most PPCM treatment modalities are based on the management of heart failure with reduced ejection fraction. Controlling volume status with diuretics is crucial, and most commonly achieved using furosemide, like with our patient, or with other loop diuretics [13]. Other agents that can be used to control volume status are nitrates, but those should be used with caution before delivery to avoid hypotension and impaired uterine perfusion. Angiotensin-converting enzyme inhibitors or angiotensin receptor blockers are also commonly used in the postpartum period but are contraindicated during pregnancy [13]. If the patient is still pregnant, a combination of nitrates and hydralazine can be used instead. Moreover, beta-blockers should be added, and are likely safe during pregnancy [13]. Past studies have suggested that prolactin may be involved in the development of PPCM, and have prompted research on prolactin inhibition with bromocriptine in PPCM. The following has also been studied in recent preclinical models where cabergoline has been shown to decrease the onset of PPCM in mice models [18]. A small prospective study randomized females with PPCM to standard therapy versus standard therapy with bromocriptine and showed modest improvement in clinical outcomes for patients treated with bromocriptine addition, including improved six-month left ventricular function [19]. Furthermore, cabergoline has also shown some benefits in the treatment of PPCM [20]. Nevertheless, due to limited data, the use of bromocriptine or cabergoline in PPCM remains questionable and not commonly employed. Breastfeeding cessation to decrease prolactin has also been considered for PPCM; however, owing to the potential side effects to the newborn from breastfeeding cessation, this management is not commonly recommended [13]. In light of the limited data on the management of PPCM, PPCM treatment remains variable and mostly adapted from the treatment of systolic heart failure.

## Conclusions

We report a rare case of PPCM with biventricular thrombosis. Our patient was treated successfully with standard medical therapy for heart failure and anticoagulation for thrombosis and clinically recovered in approximately one week. The patient continues to be followed up for any potential complications of her condition.
